# Dynamics of Minimal Residual Disease in Neuroblastoma Patients

**DOI:** 10.3389/fonc.2019.00455

**Published:** 2019-06-04

**Authors:** Suguru Uemura, Toshiaki Ishida, Khin Kyae Mon Thwin, Nobuyuki Yamamoto, Akihiro Tamura, Kenji Kishimoto, Daiichiro Hasegawa, Yoshiyuki Kosaka, Nanako Nino, Kyaw San Lin, Satoru Takafuji, Takeshi Mori, Kazumoto Iijima, Noriyuki Nishimura

**Affiliations:** ^1^Department of Pediatrics, Kobe University Graduate School of Medicine, Kobe, Japan; ^2^Department of Hematology and Oncology, Kobe Children's Hospital, Kobe, Japan

**Keywords:** neuroblastoma, minimal residual disease (MRD), cancer stem cell (CSC), circulating tumor cell (CTC), disseminating tumor cell (DTC), circulating tumor DNA (ctDNA), epithelial-mesenchymal transition (EMT)

## Abstract

Neuroblastoma is a common extracranial solid tumor of neural crest (NC) origin that accounts for up to 15% of all pediatric cancer deaths. The disease arises from a transient population of NC cells that undergo an epithelial-mesenchymal transition (EMT) and generate diverse cell-types and tissues. Patients with neuroblastoma are characterized by their extreme heterogeneity ranging from spontaneous regression to malignant progression. More than half of newly diagnosed patients present highly metastatic tumors and are stratified into a high-risk group with dismal outcome. As many as 20% of high-risk patients have residual disease that is refractory or progressive during induction chemotherapy. Although a majority of high-risk patients achieve remission, larger part of those patients has minimal residual disease (MRD) that causes relapse even after additional consolidation therapy. MRD is composed of drug-resistant tumor cells and dynamically presented as cancer stem cells (CSCs) in residual tumors, circulating tumor cells (CTCs) in peripheral blood (PB), and disseminated tumor cells (DTCs) in bone marrow (BM) and other metastatic sites. EMT appears to be a key mechanism for cancer cells to acquire MRD phenotypes and malignant aggressiveness. Due to the restricted availability of residual tumors, PB and BM have been used to isolate and analyze CTCs and DTCs to evaluate MRD in cancer patients. In addition, recent technical advances make it possible to use circulating tumor DNA (ctDNA) shed from tumor cells into PB for MRD evaluation. Because MRD can be detected by tumor-specific antigens, genetic or epigenetic changes, and mRNAs, numerous assays using different methods and samples have been reported to detect MRD in cancer patients. In contrast to the tumor-specific gene-rearrangement-positive acute lymphoblastic leukemia (ALL) and the oncogenic fusion-gene-positive chronic myelogenous leukemia (CML) and several solid tumors, the clinical significance of MRD remains to be established in neuroblastoma. Given the extreme heterogeneity of neuroblastoma, dynamics of MRD in neuroblastoma patients will hold a key to the clinical validation. In this review, we summarize the biology and detection methods of cancer MRD in general and evaluate the available assays and clinical significance of neuroblastoma MRD to clarify its dynamics in neuroblastoma patients.

## Introduction

**Figure 1:**
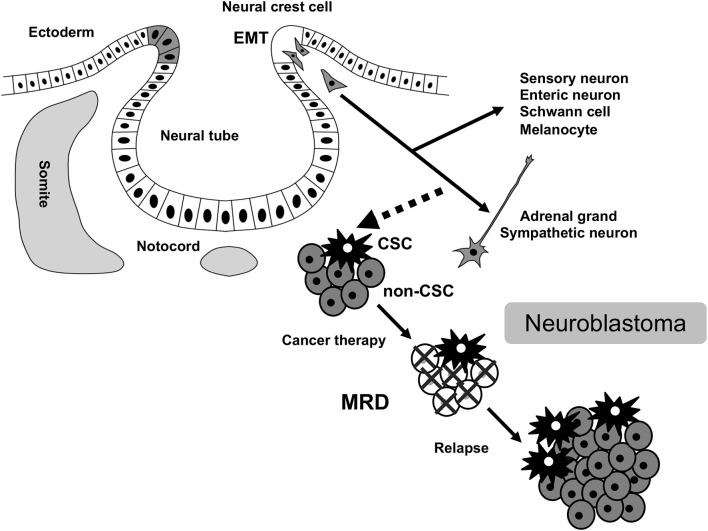
MRD in neuroblastoma. MRD, minimal residual disease; CSC, cancer stem cell; EMT, epithelial-mesenchymal transition.

Neuroblastoma is a common extracranial solid tumor in children and accounts for ~15% of all pediatric cancer-associated mortalities. The disease originates from a transient population of neural crest (NC) cells, especially a subpopulation of NC cells committed to the establishment of the sympathoadrenal tissues (Figure 1). NC cells undergo an epithelial-mesenchymal transition (EMT), and migrate throughout the developing body, and generate diverse cell-types and tissues. Various subpopulations of NC cells adopt specific migratory behaviors that has iterated in highly metastatic neuroblastoma (1, 2). EMT is central to the initiation of NC cell migration and confers on neuroblastoma cells invasive phenotype: increased tumor-initiating and metastatic potential and a greater drug-resistance (3, 4). Indeed, FoxD3, Slug, and Sox9/10 constitute the minimal inducers of EMT in NC cells and have all been shown to be dysregulated in aggressive neuroblastoma (5–7).

Patients with neuroblastoma are characterized by their extreme heterogeneity that has been described at multiple levels. These include the anatomical localization of the tumor (8), histology, genomic/molecular profile, and clinical manifestations. Histology of neuroblastoma is mainly defined by the relative proportion of neuroblastic ganglionic cells and reactive stromal Schwann-like cells that determines the tumor's differentiation status. Intratumor heterogeneity of neuroblastoma has been reported recently both at the cellular and molecular levels. These studies conjointly reported two or three types of interconvertible tumor cells with divergent gene expression profiles (undifferentiated mesenchymal and committed adrenergic cell types or NC-like, sympathoadrenal, and mixed cell types) (9, 10). Infants with neuroblastoma frequently experience spontaneous regression, whereas children more than 18-months old with neuroblastoma usually have highly metastatic and aggressive disease (11, 12).

Using a subset of these known clinical and biological prognostic factors, patients with neuroblastoma are classified into three risk groups: low-risk, intermediate-risk, and high-risk. For patients with low-risk and intermediate-risk neuroblastoma, this risk stratification, and subsequent treatment has been successful with over 90% long-term event-free survival rates (13, 14). However, treating high-risk neuroblastoma remains a significant challenge despite intensive multimodal treatment. Children with high-risk neuroblastoma account for approximately half of newly diagnosed patients and are currently treated by the standard regimens containing four main components: induction chemotherapy, local control (surgery and radiation therapy), consolidation therapy, and maintenance therapy (13, 14). Overall survival of high-risk neuroblastoma has improved from 29 to 50% over the past 20 years, mainly due to the intensification of therapy through myeloablative therapy and immunotherapy (15). Furthermore, a >70% of long-term remission is suggested for high-risk patients who received all planned therapy of tandem myeloablative therapy with autologous stem cell transplants and immunotherapy in a recent Children's Oncology Group study ANBL0532 (16). Despite these progress, as many as 20% of high-risk patients have residual disease that is refractory or progressive during induction chemotherapy. The rest of high-risk patients usually can achieve remission, but larger part of those patients has minimal residual disease (MRD) that causes relapse even after additional consolidation therapy. Patients with relapsed neuroblastoma can rarely be cured with < 10% of long-term survival (13, 14).

MRD is defined as drug-resistant persistent tumor cells following cancer therapy. The accurate and sensitive detection of MRD is essential to achieve optimal outcome in neuroblastoma patients (15, 17). Accordingly, numerous MRD assays using different methods and samples have been reported over past two decades but their clinical significance remains to be established. Given the extreme heterogeneity of neuroblastoma, understanding the dynamics of MRD in neuroblastoma patients will be critical to validate these MRD assays. In this review, we will first overview the biology and detection methods of cancer MRD in general and then examine the available assays and clinical significance of neuroblastoma MRD to clarify its dynamics in neuroblastoma patients.

## Minimal Residual Disease (MRD)

### Dynamics of MRD

MRD is conceptually defined as residual tumor cells that persistently reside in patients following local and systemic cancer therapy, and its activation causes tumor metastasis and relapse that continue to represent the most difficult challenges for cancer patients (18, 19). Most methods used in the current clinical practices for tumor detection are based on imaging studies and tumor marker assays. Early identification of tumor metastasis and relapse is critical to achieve optimal outcome since therapeutic intervention is more effective and successful in treating smaller tumor. This has led to an intensive research to develop more sensitive and accurate methods for the assessment of MRD status (20).

**Figure 2:**
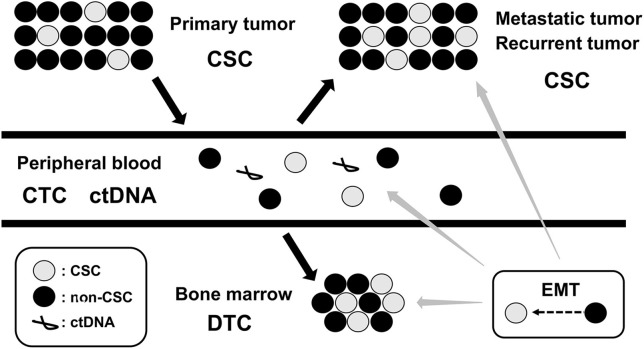
Dynamics of MRD. MRD, minimal residual disease; CSC, cancer stem cell; CTC, circulating tumor cell; DTC, disseminating tumor cell; ctDNA, circulating tumor DNA; EMT, epithelial-mesenchymal transition.

Cancer cells in primary tumor can often gain the ability to invade, migrate, disseminate, and proliferate in distant locations, representing precursors of metastasis and relapse (21). Following local and systemic cancer therapies, residual cancer cells persist as cancer stem cells (CSCs) in primary tumor, circulating tumor cells (CTCs) in peripheral blood (PB), and disseminated tumor cells (DTCs) in bone marrow (BM), lymph node, and micrometastasis in other metastatic tissues. These CSC, CTC, and DTC represent the dynamics of MRD in cancer patients (22) (Figure 2). Since invasive tumor biopsies of primary, metastatic, and recurrent tumors cannot always be performed, the detection and analysis of CTC and DTC by less invasive sampling of PB and BM has been shown to be of clinical relevance in many cancer types, particularly in breast, colon, and prostate cancers (23–25). In addition, circulating tumor DNA (ctDNA), which is shed from tumor cells into PB and isolated from blood serum or plasma, has also been used for diagnostic and prognostic purposes in several cancer types (26, 27). The detection and analysis of CTC, DTC, and ctDNA will serve as complementary methods for the assessment of MRD status in cancer patients (Figure 2).

### Cancer Stem Cell (CSC)

Tumors are composed of multiple phenotypically distinct subpopulations of cancer cells, which is considered a major driver of the development of resistance to cancer therapy (28, 29). Genome sequencing has delineated the types of genetic changes underling the phenotypic alteration and diversification of cancer cells (30). The ability of cancer cells to frequently interconvert between multiple alternative phenotypic states without genetic changes (epigenetic changes) is also increasingly recognized (31, 32). CSC has provided key insights into how epigenetic mechanisms can contribute to the phenotypic diversity of cancer cells within a tumor (33, 34). Over the past decades, numerous studies have suggested that only a small subpopulation of the cancer cell with tumor-initiating capability, named as CSC, is the core origin of the tumorigenesis. CSCs are defined as cancer cells that have the abilities to self-renew and differentiate into their progeny (non-CSCs). Functionally, CSCs show tumor-initiating potential *in vivo*, anchorage-independent growth *in vitro*, and drug-resistance. Methodologically, CSCs can be isolated from tumor samples using flow cytometry employing cell-surface markers such as CD44 and CD133, or functional characteristics such as dye extrusion capability (side population), aldehyde dehydrogenase (ALDH) activity, and sphere formation capability (35). The clinical relevance of CSCs is typically seen in metastatic and recurrent tumors, which are enriched with CSCs and associated with CSC-gene signatures (36).

**Figure 3:**
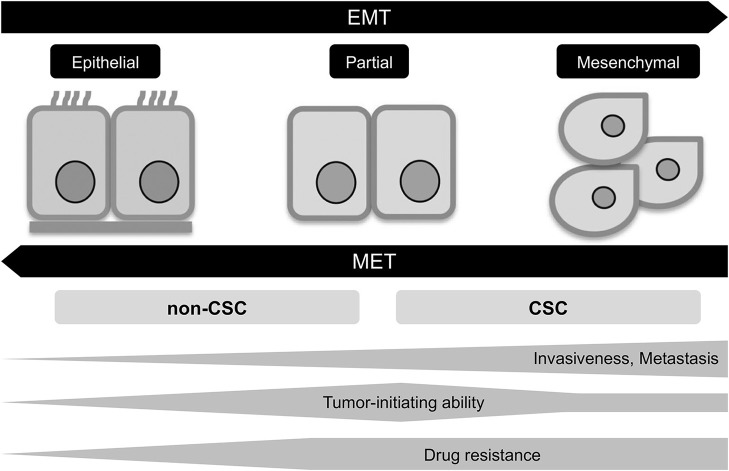
EMT program in tumor progression. EMT, epithelial-mesenchymal transition; MET, mesenchymal-epithelial transition; CSC, cancer stem cell.

The epigenetic changes between CSCs and non-CSCs seems to be linked to a distinct transcriptional program found during developmental tissue remodeling referred to as EMT, which imparts heritable phenotypic changes to cancer cells through epigenetic modifications without introducing new genetic changes (37) (Figures 2, 3). Upon activation of EMT program, cancer cells lose many of their epithelial characteristics including epithelial cell junctions and apical–basal polarity, and instead acquire mesenchymal attributes such as an increased capacity for migration and invasion (38). In a number of cancer types, only cancer cells within CSC-enriched subpopulations exhibit aspects of activated EMT-program. In addition, EMT-program activation increases the capacity of tumor initiation and confers resistance to various therapeutic agents (3, 4, 39). The CSC phenotypes (CSCs or non-CSCs) interconnected to activated EMT-program likely define a continuum of cellular states, leading to the distinct subpopulations of CSCs (40) (Figures 2, 3).

### Circulating Tumor Cell (CTC)

CTC represents an intermediate of tumor metastasis that requires the acquisition of diverse properties including invasion and migration from the primary tumor, intravasation, survival, extravasation, colonization at a distinct tissues, and growth into a macro metastatic lesion (41, 42). Direct evidences for metastatic precursors of CTCs have recently came from breast and lung cancers that is characterized by early dissemination and dismal prognosis (43, 44). While some CTCs passively enter the bloodstream, CTCs derived from actively invading tumor cells can acquire EMT and CSC phenotypes required for tumor metastasis (45, 46). CTCs can circulate as single cells or clusters, with clusters having increased metastatic potential and a shorter half-life in the circulation (6–10 min for clusters and 25–30 min for single cells) (47). Most CTCs die in the circulation, whereas the surviving CTCs either extravasate into the adjacent tissue or become lodged in the capillary beds of homing tissues (48).

While CTCs are a very rare population of PB sample, they are accessible through simple non-invasive PB sampling. In metastatic tumors, CTCs are derived from both the primary and metastatic tumors, and only from the metastatic tumors when the primary tumor has been resected. Among independent metastatic tumors within a single patient, each tumor can evolve independently and acquire *de novo* mutations (41, 42). Since surgical resections and biopsies of metastatic tumors are prone to have sampling bias and limited landscape of mutations across all metastatic tumors, CTCs may provide less-biased sampling and more global picture of metastatic tumors. The recent advances in technology for the enrichment and characterization of CTCs have begun to provide new insight into the mechanisms of tumor metastasis and relapse (25, 49).

### Disseminated Tumor Cell (DTC)

Although CTCs provide a valuable information about the aggressiveness of tumor, there are additional barriers for CTCs to gain the ability to form metastatic tumors (41, 42). Once CTCs has entered the bloodstream, they must survive, exit the bloodstream, and grow in a foreign microenvironment to cause metastasis. Survived CTCs in the bloodstream disseminate into homing tissues, such as BM, lymph nodes, lung, liver, and brain, and called DTCs (25, 49). Although DTCs and CTCs present similar cytologic and membrane markers, they represent two distinct phenotypes: epithelial-type DTCs and mesenchymal-type CTCs. While a small minority of DTCs generates a proliferating metastatic tumor, a majority of DTCs spread throughout the parenchyma of homing tissues. Depending on cancer types, metastatic tumors develop over a period of months to years (50). A direct contribution of DTCs to metastases formation have shown in breast and esophageal cancers. Presence of DTCs at the time of primary tumor diagnosis or following systemic treatment strongly correlated with metastasis at distant sites in breast cancer patients (51). DTCs established from micrometastatic lymph nodes of esophageal cancer patients have generated tumors in immune-compromised mice (52).

DTCs have been found long after curative treatment of tumor, even in patients without overt metastasis. These DTCs may enter a dormant state but retain the ability to grow into a metastatic tumor or may reach an equilibrium between cellular proliferation and cell death or elimination. Dormant DTCs remain clinically silent and are apparently maintained by an interaction with microenvironment in their homing tissues (53, 54). In breast and prostate cancers, dormant DTCs have acquired additional genetic and epigenetic changes or significant alteration in their microenvironment, and resulted in very late relapses that occur years after treatment (55). BM is the most common homing tissue for blood borne DTCs derived from primary and metastatic tumors. As with CTCs, studies of DTCs in BM have apparent advantages over other homing tissues, which include accessibility, analysis through flow cytometry, and opportunity to gain real-time information about tumors (25, 49).

### Circulating Tumor DNA (ctDNA)

Circulating tumor DNA (ctDNA) is a fraction of extracellular nucleic acid (cell-free DNA) that is released into the bloodstream by tumor cells. While CTCs are isolated from the cell pellet containing normal blood cells, ctDNA is isolated from the supernatant. Because the number of CTCs is very low in PB, ctDNA is more sensitive in detecting tumor-associated genetic and epigenetic changes in tumors (26, 27). Although ctDNA can be isolated both from plasma and serum, plasma is recommended as a preferred source for ctDNA because serum-derived ctDNA is contaminated with normal blood cell DNA during the clotting process (56). The release of ctDNA into PB is caused by the apoptosis, necrosis, autophagy, secretion, and other mechanisms, but the exact mechanism of ctDNA release remains unclear (57). In PB, ctDNA circulates predominantly in the form of nucleosomes that retains at least some features of the nuclear chromatin and enables the determination of genetic and epigenetic changes. The size of ctDNA varies between small fragments of 70–200 base pairs and large fragments of ~21 kilobases (26). ctDNA is cleared from the blood by the kidney, liver, and spleen and has a variable half-life ranging from 15 min to several hours (58).

Although cancer patients have higher ctDNA levels than healthy control individuals, the concentrations of overall ctDNA vary considerably in plasma or serum samples in both groups. In cancer patients, ctDNA concentrations are generally lower in localized tumors and higher in metastatic tumors, but also affected by tumor burden, vascularity, and treatment response (56). Tumor-associated genetic and epigenetic changes such as point mutations, genomic rearrangements, copy number variation (CNV), microsatellite instability (MSI), loss of heterozygosity (LOH), and DNA methylation have been detected in ctDNA. These changes warrant a distinction between ctDNA and remaining cell-free DNA, and allow the use of ctDNA as potential biomarkers for determining tumor state, tumor metastasis and relapse, and treatment response (27).

## MRD Detection Methods

### Cell-Based Assays

While conventional cytology and histology have been accepted as the gold standard for initial diagnosis and staging of tumor patients, these methods are not always possible to detect tumor cells below the level of 1% by morphology alone (59, 60). To achieve more sensitive and quantitative detection of MRD, single-cell-based analytical methods of immunocytology (IC), and flow cytometry (FCM) are applied for quantification of MRD in BM and PB samples (61–63). Both IC and FCM methods rely on the availability of antibodies to tumor-associated cell-surface or intracellular antigens that are ideally expressed on all tumor cells but not normal hematopoietic cells. The sensitivity of IC solely depends on the number of investigated cells and reaches a single tumor cell in 10^4^-10^5^ normal cells in most clinical settings, whereas the sensitivity of FCM is about 10 times lower than IC due to the requirement for at least 10–20 events to call a sample positive (64–66).

### gDNA-Based Assays

Tumor DNA can be detected by tracking tumor-specific changes in CTCs, DTCs, and ctDNA samples. In contrast to cell-based assays that are dependent on the availability of antibodies, gDNA-based assays have relied on the existence of tumor-specific genetic and epigenetic changes such as point mutations, genomic rearrangements, CNV, MSI, LOH, and DNA methylation (20, 67). Polymerase chain reaction (PCR) amplification of genomic rearrangements has been particularly powerful in the characterization and detection of hematopoietic tumors. In acute lymphoblastic leukemia (ALL), tumor cells can be detected by PCR amplification of leukemia-specific junctional regions of rearranged immunoglobulin (Ig) genes, and T-cell receptor (TCR) genes (68, 69). Detection of MRD in ALL patients is clinically relevant both in primary and relapsed ALL, and incorporated into patient's risk stratification (70, 71). For solid tumors, DNA sequencing of known oncogene or tumor suppressor mutations has utilized to detect tumor cells. In colorectal cancers, KRAS, APC, and TP53 have a high mutation frequency and their mutation status is correlated with diagnosis, prognosis, and treatment response (72). Recent progress in array comparative genomic hybridization (aCGH) and massively paralleled sequencing (MPS) has begun to realize the detection of patient-specific genetic and epigenetic changes rather than tumor-specific ones (73).

### mRNA-Based Assays

Although tumor RNA can be isolated from CTCs and DTCs, the detection of tumor-specific mRNA has been limited to the oncogenic-fusion gene-positive tumors. In chronic myelogenous leukemia (CML), the detection of leukemia-specific BCR-ABL fusion mRNA is used to monitor MRD, and CML patients are currently treated by BCR-ABL fusion kinase inhibitors based on the level of MRD (74, 75). However, the oncogenic-fusion genes have not been identified for most tumors. In melanoma, tumor cells have been identified by the detection of mRNA specific to the tissue of tumor origin (tumor-associated mRNA), tyrosinase mRNA (76). Since the first demonstration in melanoma, the detection of tumor-associated mRNA expression by reverse transcriptase-PCR (RT-PCR) has been applied to detect tumor cells in a number of different tumors. The value of the detection of tumor-associated mRNA expression by RT-PCR has been dependent on the samples evaluated (PB, BM, or lymph nodes), the tumor type, and the clinical stage of tumor (20).

## MRD Detection in Neuroblastoma Patients

### Neuroblastoma-Associated Antigen Detection

**Figure 4:**
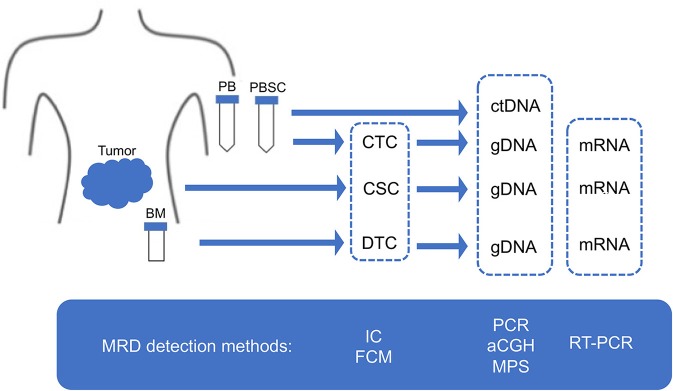
MRD detection methods. MRD, minimal residual disease; CSC, cancer stem cell; CTC, circulating tumor cell; DTC, disseminating tumor cell; ctDNA, circulating tumor DNA; PB, peripheral blood; PBSC, peripheral blood stem cell; BM, bone marrow; IC, immunocytology; FCM, flow cytometry; PCR, polymerase chain reaction; aCGH, array comparative genomic hybridization; MPS, massively paralleled sequencing; RT-PCR, reverse transcriptase-PCR.

**Figure 5:**
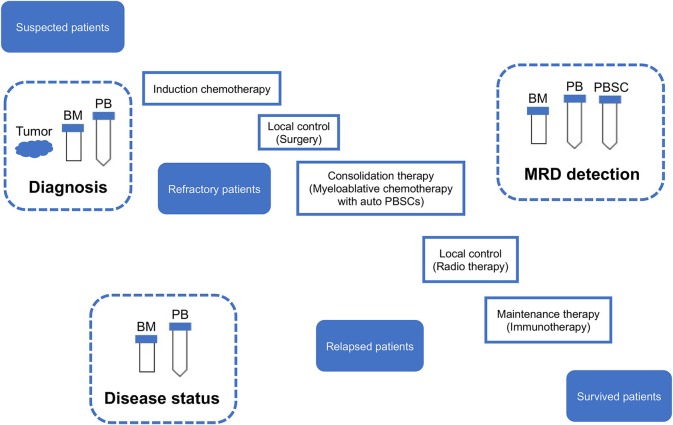
Sampling from neuroblastoma patients. MRD, minimal residual disease; PB, peripheral blood; PBSC, peripheral blood stem cell; BM, bone marrow; IC, immunocytology.

Given the limitation of conventional morphology, single-cell-based IC and FCM with anti-neuroblastoma-associated antigen antibodies have been used to detect residual neuroblastoma cells in BM, PB, and peripheral blood stem cell (PBSC) samples (77–79). A variety of antibodies raised against neuroblastoma cells and established neuronal markers such as neuron specific enolase (NSE) (80), synaptophysin (81), NB84 (82), chromogranin A (83), and CD56 (N-CAM) (84, 85) have been tested for IC. However, many of these antibodies are inappropriate for MRD detection due to their heterogeneous labeling in individual tumors, cross-reactivity with normal hematopoietic cells, or differential labeling on primary vs. metastatic and on undifferentiated vs. differentiated tumor cells (86). Among these neuroblastoma-associated antigens, a cell surface glycosphingolipid, disialoganglioside (GD2), is homogeneously and strongly expressed on neuroblastic tumors but not on normal hematopoietic cells (78, 87, 88). Although the reliability of tumor cell detection and quantification by IC had been controversial, IC detection of neuroblastoma cells was standardized in BM aspirates by using fixed cytospins and anti GD2-antibody (63) and recommended for single-cell based sensitive MRD detection in BM, PB, and PBSC samples by the International Neuroblastoma Risk Group (INRG) (66) (Figures 4, 5, Table 1).

**Table 1:** Comparison of neuroblastoma MRD detection methods.

**Methods**	**Targets/markers**	**Application**
IC	GD2, NSE, synaptophysin, NB84, chromogranin A, CD56 (N-CAM)	BM, PB, PBSC (Sample preparation) Fixed cytospins (Advantage) Gold standard (Disadvantage) Low sensitivity, Inconsistency (Applicability) Depends on the availability of antibody
FCM	CD9+/CD56+/CD45–, CD81+/CD56+/CD45–, GD2+/CD56+/CD45–, NB84+/CD56+/CD45–, GD2+/CD81+/CD56+/CD45–, CD9+/CD81+/CD56+/CD45–	BM, PB, PBSC (Sample preparation) Dissociated cell suspension (Advantage) Consistency (Disadvantage) Low sensitivity (Applicability) Depends on the availability of antibody
PCR aCGH MPS	Neuroblastoma-specific genetic and epigenetic changes Patient-specific genetic and epigenetic changes	BM, PB, PBSC (Sample preparation) ctDNA, gDNA (Advantage) High sensitivity, Consistency (Disadvantage) Difficulty in identifying the genetic and epigenetic changes (Applicability) Depends on the availability of genetic and epigenetic changes
RT-PCR	Neuroblastoma-associated mRNAs	BM, PB, PBSC (Sample preparation) mRNA (Advantage) High sensitivity (Disadvantage) Inconsistency (Applicability) No restriction

Although immunophenotyping with FCM has proven to be essential for differential diagnosis of most hematopoietic tumors, it remains optional for pediatric solid tumors including neuroblastoma (89). Neuroendocrine tumors were reported to display CD56+/CD45– immunophenotype (90). In addition to CD56, CD9, CD81, GD2, and NB84 antigens were used for FCM. The first multiparameter FCM application for neuroblastoma cell detection was based on CD9+/CD56+/CD45– immunophenotype (61), which was later replaced by CD81+/CD56+/CD45– immunophenotype (91). Subsequently, GD2+/CD56+/CD45–, NB84+/CD56+/CD45–, GD2+/CD81+/CD56+/CD45–, and CD9+/CD81+/CD56+/CD45– immunophenotypes were also applied to FCM-based MRD detection (64, 65, 92, 93) (Figures 4, 5, Table 1). However, a very limited data about prognostic value are currently available for FCM-based MRD detection methods. Detection of NB84+/CD56+/CD45– cells by FCM in initial diagnostic BM samples from metastatic neuroblastoma patients had predicted significantly poor cumulative survival (93). Neuroblastoma patients with GD2+/CD81+/CD56+/CD45– cells in BM by FMC had significantly worse event-free survival and increased cumulative incidence of relapse/progression (94) (Table 4).

### Neuroblastoma-Specific gDNA Detection

While tumor DNA can be detected by tracking tumor-specific genetic and epigenetic changes in CTCs, DTCs, and ctDNA samples, neuroblastoma-specific genetic changes such as known oncogene or tumor suppressor mutations and genomic rearrangements have not been identified yet (102). For epigenetic changes specific to neuroblastoma, DNA methylation of tumor suppressor RASSF1A has been applied to detect MRD in DTCs samples (103). In addition to neuroblastoma-specific genetic and epigenetic changes, patient-specific ones have begun to be identified in DTCs and ctDNA sample. Patient-specific DNA breakpoints in eight high-risk neuroblastoma patients have been identified by whole genome sequencing (WGS) of primary tumor and used to detect MRD in DTCs samples (104). Patient-specific genomic mutations in 19 neuroblastoma patients have been detected by whole exome sequencing (WES) of sequentially collected ctDNA samples, revealing a shift of mutational pattern over time and a generation of treatment-resistant clones (105) (Figures 4, 5, Table 1).

### Neuroblastoma-Associated mRNA Detection

#### Neuroblastoma-Associated mRNAs

A number of neuroblastoma-associated mRNAs have been identified initially as a single marker (Table 2) and later as a set of multiple markers (Table 3) for qPCR-based MRD detection in neuroblastoma patients.

**Table 2:** Neuroblastoma-associated mRNAs.

**Gene symbol**	**Gene name**	**References**
*B4GALNT1*	Beta-1,4-N-acetyl-galactosaminyl transferase 1GD2-synthase (GD2-s)	(106)
*CCND1*	Cyclin D1	(107)
*CHGA*	Chromogranin A (CGA)	(108)
*CHGB*	Chromogranin B Secretogranin 1 (SCG1)	(109)
*CHRNA3*	Cholinergic receptor nicotinic alpha 3 subunit	(109)
*CRMP1*	Collapsing response mediator protein 1	(110)
*DBH*	Dopamine beta-hydroxylase	(111)
*DCX*	Doublecortin	(112)
*DDC*	Dopa decarboxylase	(113)
*ELAVL4*	ELAV like RNA binding protein 4	(114)
*GABRB3*	Gamma-aminobutyric acid type A receptor beta 3 subunit	(110)
*GAGE*	G antigen	(115)
*GAP43*	Growth associated protein 43	(109)
*ISL1*	ISL LIM homeobox 1	(110)
*KIF1A*	Kinesin family member 1A	(110)
*PHOX2B*	Paired-like homeobox 2b	(116)
*SNAP91*	Synaptosome associated protein 91	(109)
*ST8SIA2*	ST8 alpha-N-acetyl-neuraminide alpha-2,8-sialyltransferase 2 sialyltransferases (STX)	(117)
*STMN2*	Stathmin 2	(109)
*STMN4*	Stathmin 4	(109)
*TACC2*	Transforming, acidic coiled-coil containing protein 2	(110)
*TH*	Tyrosine hydroxylase	(111, 118, 119)
*UCHL1*	Ubiquitin C-terminal hydrolase L1 Neuron cytoplasmic protein 9.5 (PGP9.5)	(120)

**Table 3:** Sets of neuroblastoma-associated mRNAs.

**Marker genes**	**Reference genes**	**Sample**	**References**
*CCND1, DDC, GABRB3, ISL1, KIF1A, PHOX2B*	*B2M*	BM	(110)
*DCX, PHOX2B, TH*	*B2M*	BM Ovary Testis	(121–123)
*CHRNA3, DDC, GAP43, PHOX2B, TH*	*GUSB*	BM	(109)
*CHRNA3, DBH, DDC, PHOX2B, TH*	*GUSB*	PB PBSC	(109, 124)
*CHRNA3, CRMP1, DBH, DCX, DDC, GABRB3, GAP43, ISL1, KIF1A, PHOX2B, TH*	*B2M*	BM, PB	(125)
*CHGA, DCX, DDC, PHOX2B, TH*	*B2M, GAPDH, HPRT1, SDHA*	PBSC BM, PB	(101, 126)
*B4GALNT1, DDC, TH*	Calibrator	BM, PB	(127, 128)
*DCX, TH*	*GAPDH*	BM, PB	(95)
*B4GALNT1, CCND1, ISL1, PHOX2B*	*B2M*	BM	(129, 130)
*B4GALNT1, ELAVL4, PHOX2B, TH*	*ABL*	BM	(131)

In the absence of tumor-specific mRNA, the rate-limiting enzyme in catecholamine biosynthesis, TH, was evaluated as the first neuroblastoma-associated mRNA to detect MRD in neuroblastoma patients (132). While amplification of TH mRNA was shown to detect neuroblastoma cells in BM and PB samples (118, 119), low level of its expression in normal BM cells initially limited its specificity (111). Application of quantitative RT-PCR (qPCR) made it possible to quantify the number of TH mRNA transcripts and allowed to set a threshold of its expression between tumor and normal cells for neuroblastoma MRD detection in BM and PB samples. A number of additional neuroblastoma-associated mRNAs were then reported to detect MRD in PB and BM samples (Table 2). Among these markers, PHOX2B was identified as the most specific and sensitive marker for qPCR-based MRD detection in neuroblastoma patients (116). However, PHOX2B was not highly expressed in all neuroblastoma tumors and its expression considerably varied among different neuroblastoma patients (109).

Given the extreme heterogeneity of neuroblastoma, an increasing number of multiple marker sets for qPCR-based MRD detection have been reported (Table 3). A set of six markers (CCND1, DDC, GABRB3, ISL1, KIF1A, PHOX2B) was identified by genome-wide gene expression microarray analyses of 48 neuroblastoma tumors and nine remission BM samples followed by selecting genes with higher tumor-to-BM expression ratios (110). A set of three markers (DCX, PHOX2B, TH) was identified by genome-wide gene expression microarray analyses of 32 neuroblastoma tumors and pooled PB sample from 24 healthy volunteers followed by selecting genes not expressed in pooled PB samples (121–123). Two sets of five markers (CHRNA3, DDC, GAP43, PHOX2B, TH) for BM samples and five markers (CHRNA3, DBH, DDC, PHOX2B, TH) for PB samples were identified by comparing serial analysis of gene expression (SAGE) libraries of neuroblastoma and healthy tissues followed by qPCR analysis of 56 neuroblastoma tumors, 51 control BM samples, and 37 control PB samples (109, 124). A set of 11 markers (CHRNA3, CRMP1, DBH, DCX, DDC, GABRB3, GAP43, ISL1, KIF1A, PHOX2B, TH) was identified by validating known 14 makers expression in CSC-enriched spheres of neuroblastoma BE(2)-C cells (125). A set of five markers (CHGA, DCX, DDC, PHOX2B, TH) normalized by four references (B2M, GAPDH, HPRT1, SDHA) was also developed (101, 126). In addition, several amended sets of two markers (DCX, TH) for BM and PB samples (95), three markers (B4GALNT1, DDC, TH) for BM and PB samples (127), four markers (B4GALNT1, CCND1, ISL1, PHOX2B) for BM sample (129, 130), and four markers (B4GALNT1, ELAVL4, PHOX2B, TH) for BM sample were reported (131).

#### Clinical Significance of qPCR-Based MRD Detection

A growing number of qPCR-based MRD detection assays using different methods (MRD markers) and samples have been reported over past two decades. These assays use three types of samples (BM, PB, and PBSC). BM is the most common site of infiltration in metastatic neuroblastoma patients at the time of diagnosis, and is a frequent site of tumor relapse (133). Neuroblastoma cells have been detected in PB of metastatic neuroblastoma patients at diagnosis and during therapy (78). High-risk neuroblastoma patients are treated with the standard regimens containing high-dose myeloablative chemotherapy rescued with autologous stem cells (auto PBSCs) and have naturally expected to cause relapse if PBSC contaminated with neuroblastoma cells are transplanted (126). These assays are also targeted for different groups of neuroblastoma patients (localized and metastatic neuroblastoma) (Figures 4, 5, Table 1).

Accordingly, qPCR-based MRD detection assays can be classified into the following five groups: (1) Localized neuroblastoma-PB samples, (2) Localized neuroblastoma-BM samples, (3) Metastatic neuroblastoma-PBSC samples, (4) Metastatic neuroblastoma-PB samples, (5) Metastatic neuroblastoma-BM samples. At the moment, the clinical significance of all five groups remains to be established. However, the promising data has been reported in two groups (Metastatic neuroblastoma-PB and BM samples).

##### Localized neuroblastoma-PB and BM samples

In localized neuroblastoma, the clinical significance of qPCR-based MRD detection is still not clear in both PB and BM samples.

Expression of B4GALNT1, DCX, DDC, ELAVL4, PHOX2B, ST8SIA2, and TH mRNAs in BM and PB samples at diagnosis was not associated with clinical events in localized neuroblastoma patients (134). MRD detection evaluated by DCX or TH mRNA expression in PB samples at diagnosis predicted worse event-free survival (EFS) of non-metastatic neuroblastoma patients (95). When BM samples are evaluated by a set of five markers (CHRNA3, DDC, GAP43, PHOX2B, and TH), unfavorable outcome in localized neuroblastoma patients is associated with the detection of more than one positive-marker (99) (Table 4).

**Table 4:** Prognostic significance of neuroblastoma MRD detection methods.

**Methods**	**Targets**	**Samples and patients**	**Outcome**	**References**
IC	GD2	PB Patients during chemotherapy	MRD detection was significantly correlated with relapse.	(78)
FCM	NB84+/CD56+/CD45–	BM Stage 4 patients at diagnosis	MRD detection was significantly correlated with lower OS.	(93)
FCM	GD2+/CD81+/CD56+/CD45–	BM Patients at diagnosis	MRD detection was significantly correlated with lower EFS.	(94)
RT-PCR	DCX, TH	BM, PB Non-metastatic patients at diagnosis	MRD detection in PB was significantly correlated with lower EFS.	(95)
RT-PCR	CHRNA3, DDC, GAP43, PHOX2B, TH	BM Stage 4 and over 1-year patients at 3 months after diagnosis and after induction chemotherapy	MRD detection was significantly correlated with poor outcome.	(96)
RT-PCR	TH	PBSC High-risk patients	MRD detection was significantly correlated with lower 2-year OS.	(97)
RT-PCR	DCX, PHOX2B, TH	BM, PB High-risk patients at diagnosis and after induction chemotherapy	High levels of DCX, PHOX2B, or TH mRNAs in BM and PB at diagnosis or in BM after induction therapy predicted significantly poor outcome.	(98)
RT-PCR	CHRNA3, DDC, GAP43, PHOX2B, TH	BM Localized patients at diagnosis	MRD detection with more than one marker was significantly associated with lower EFS.	(99)
RT-PCR	DCX, TH	BM, PB Metastatic patients at diagnosis, after induction therapy, and at the end of treatment	High levels of DCX mRNAs in BM and PB at diagnosis or in BM after the induction therapy predicted significantly poor outcome. High levels of TH mRNAs in BM and PB at diagnosis, in PB after the induction therapy, or in PB at the end of treatment predicted significantly poor outcome.	(100)
RT-PCR	CHGA, DCX, DDC, PHOX2B, TH	BM, PB High-risk patients with relapsed/progressive disease or refractory disease	Levels of 5NB-mRNAs was significantly correlated with PFS.	(101)

##### Metastatic neuroblastoma-PBSC samples

In metastatic neuroblastoma, the clinical significance of qPCR-based MRD detection is still not clear in PBSC samples.

There are conflicts between the previous reports. TH mRNA expression in PBSC samples was not statistically associated with unfavorable outcome in high-risk metastatic neuroblastoma patients (135). qPCR-based MRD detection by B4GALNT1 or TH mRNAs in PBSC samples did not affect survival of stage 4 neuroblastoma patients (136). High-risk neuroblastoma patients received unpurged PBSCs did not have more relapses (126). In contrast to these reports, positive TH mRNA in PBSC samples predicted a lower 2-year OS in high-risk neuroblastoma patients (97). High levels of CHGA, DCX, DDC, PHOX2B, or TH mRNA expression in PBSC samples was associated with worse outcome (126) (Table 4).

##### Metastatic neuroblastoma-PB and BM samples

In metastatic neuroblastoma, the clinical significance of qPCR-based MRD detection remains to be established in both PB and BM samples.

While the independent study groups reported the promising data, they used different methods (MRD markers) and their data remained controversial. MRD detection in BM samples evaluated by a set of five markers (CHRNA3, DDC, GAP43, PHOX2B, and TH) was associated with poor outcome in patients aged over 1 year with stage 4 neuroblastoma (96). High levels of DCX, PHOX2B, or TH mRNAs in BM and PB samples at diagnosis or BM samples after induction therapy predicted poor outcome in children with stage 4 neuroblastoma (98). High levels of DCX and TH mRNAs in BM and PB samples at diagnosis was associated with poor outcome in metastatic neuroblastoma (100). Expression levels of B4GALNT1, CCND1, ISL1, or PHOX2B mRNAs in BM samples predicted progression-free survival (PFS) and overall survival (OS) for anti-GD2 antibody-treated patients in first or second remission or with refractory disease (130). The combined signature of five markers (CHGA, DCX, DDC, PHOX2B, and TH) expression in BM and PB was correlated with PFS of relapsed/refractory neuroblastoma (101) (Table 4).

## Conclusion

Drug-resistant MRD is principally responsible for dismal outcome for aggressive cancers. Current intensive multimodal therapies eliminate the bulk population of tumor cells while sparing the minor subpopulations of MRD that is dynamically presented as CSCs, CTCs, and DTCs in cancer patients. EMT appears to be a key mechanism operated in cancer cells to acquire these MRD phenotypes. Although qPCR of tumor-associated mRNAs in BM and PB samples have detected clinically relevant tumor cells in many hematopoietic and solid tumors, current qPCR-based assays for neuroblastoma MRD detection use different neuroblastoma-associated mRNAs (alone or in combination) and will require the prospective quality-controlled clinical studies that evaluate the clinical significance of these qPCR-based assays. Given the extreme heterogeneity of neuroblastoma, the normal variations, which can arise from sampling of the different patients or sequential sampling of the same patient, hold a key to interpret the data from these clinical studies.

Recent advances in techniques to isolate and detect rare DNA changes in a complex mixture of DNA with high sensitivity have made it possible to track tumor-specific genetic and epigenetic changes in CTCs, DTCs, and ctDNA. Because neuroblastoma relapses often occur at multiple anatomic sites and distinct DNA aberrations can be unique to each metastatic site, the assessment of ctDNA would provide unprecedented insights into the metastatic and recurrent processes of neuroblastoma. The complementary assessment of MRD status in CTCs, DTCs, and ctDNA collected from the same patient during entire course of treatment will provide our foundation to understand the dynamics of MRD in neuroblastoma patients. Toward the optimal stratification and outcome of neuroblastoma patients, there is an urgent need for these clinical studies realizing a solid evaluation of neuroblastoma MRD.

## Author Contributions

SU, DH, YK, KI, and NoN conceptualized the manuscript. SU and NoN wrote the draft. SU, TI, KT, NY, AT, KK, NaN, KL, ST, and TM reviewed and edited the manuscript. DH, YK, KI, and NoN revised the manuscript.

### Conflict of Interest Statement

NN received an institutional research funding from the Sysmex Corporation. The remaining authors declare that the research was conducted in the absence of any commercial or financial relationships that could be construed as a potential conflict of interest.
